# 4T1 Murine Mammary Carcinoma Cells Enhance Macrophage-Mediated Innate Inflammatory Responses

**DOI:** 10.1371/journal.pone.0133385

**Published:** 2015-07-15

**Authors:** Laurence Madera, Anna Greenshields, Melanie R. Power Coombs, David W. Hoskin

**Affiliations:** 1 Department of Microbiology & Immunology, Dalhousie University, Halifax, Nova Scotia, Canada; 2 Department of Pathology, Dalhousie University, Halifax, Nova Scotia, Canada; 3 Department of Surgery, Dalhousie University, Halifax, Nova Scotia, Canada; National Cancer Institute (INCA), BRAZIL

## Abstract

Tumor progression and the immune response are intricately linked. While it is known that cancers alter macrophage inflammatory responses to promote tumor progression, little is known regarding how cancers affect macrophage-dependent innate host defense. In this study, murine bone-marrow-derived macrophages (BMDM) were exposed to murine carcinoma-conditioned media prior to assessment of the macrophage inflammatory response. BMDMs exposed to 4T1 mammary carcinoma-conditioned medium demonstrated enhanced production of pro-inflammatory cytokines tumor necrosis factor α, interleukin-6, and CCL2 in response to lipopolysaccharide (LPS) while production of interleukin-10 remained unchanged. The increased LPS-induced production of pro-inflammatory cytokines was transient and correlated with enhanced cytokine production in response to other Toll-like receptor agonists, including peptidoglycan and flagellin. In addition, 4T1-conditioned BMDMs exhibited strengthened LPS-induced nitric oxide production and enhanced phagocytosis of *Escherichia coli*. 4T1-mediated augmentation of macrophage responses to LPS was partially dependent on the NFκB pathway, macrophage-colony stimulating factor, and actin polymerization, as well as the presence of 4T1-secreted extracellular vesicles. Furthermore, peritoneal macrophages obtained from 4T1 tumor-bearing mice displayed enhanced pro-inflammatory cytokine production in response to LPS. These results suggest that uptake of 4T1-secreted factors and actin-mediated ingestion of 4T1-secreted exosomes by macrophages cause a transient enhancement of innate inflammatory responses. Mammary carcinoma-mediated regulation of innate immunity may have significant implications for our understanding of host defense and cancer progression.

## Introduction

The complex interplay between cancer cells and the host immune response is of paramount importance in the progression of breast cancer. In brief, many cancers can subvert the functions of cells within the tumor microenvironment to limit cancer-specific cell-mediated immune responses and to promote angiogenesis[1,2]. Through these processes, tumor-associated fibroblasts and other cells drive the growth of breast cancer cells and aid in the development of metastatic disease. Macrophages, as key regulators of host immunity, have profound effects on the tumor microenvironment. Tumor-associated macrophages (TAMs) are a major source of vascular endothelial growth factor and a range of matrix metalloproteinases, factors that are essential for the development of tumor vasculature[3,4]. In addition, TAMs, which display some characteristics of alternatively activated M2 macrophages, exert a localized immunosuppressive influence through an increased secretion of anti-inflammatory cytokines, including interleukin-10 (IL-10) and transforming growth factor-β (TGF-β), and the subsequent development of an inappropriate Th2-type adaptive immune response[4,5]. This crippling of cytotoxic cell-mediated responses results in severely deficient anti-tumor responses. Furthermore, the production of monocyte/macrophage chemoattractants by both tumor cells and macrophages results in the infiltration and differentiation of additional TAMs[6,7]. Indeed, the presence of TAMs is strongly correlated with poor prognoses in breast cancer patients and remains a major obstacle to effective immune-based treatment of breast cancer[8,9].

In recent years, the influence of TAMs on lymphocyte-mediated immune responses has been extensively investigated. In comparison, the effect of tumor-conditioning on macrophage-mediated responses to pathogens and, thus, overall host microbial defense, is not well understood. This is especially important as microbial motifs, such as Toll-like receptor (TLR) agonists, are being studied as components of therapeutic vaccines to counter the immune suppression of the tumor microenvironment[10]. The use of pro-inflammatory agonists against breast cancers may be seen as a double-edged sword since TLR-mediated signaling, particularly through the NFκB pathway, plays a major role in the development and progression of breast cancers[11]. Nevertheless, inflammation-promoting agonists such as imiquimod and CpG oligonucleotide (ODN) are able to inhibit tumor growth by inducing T-lymphocyte- and NK-cell-mediated anti-tumor responses[12–14].

In this study, we investigated the innate immune responses of cancer cell-conditioned macrophages to several bacterial agonists. Murine bone marrow-derived macrophages (BMDMs) were briefly conditioned with medium from different mouse carcinoma cell cultures. We observed that BMDM conditioned with 4T1 murine mammary carcinoma culture medium displayed an increased production of monocyte chemoattractant CCL2 and promoted macrophage chemotaxis, which was in concordance with other studies[15]. Importantly, our studies are the first to demonstrate that BMDM uptake of released factors from 4T1 mammary carcinomas lead to an increase in macrophage inflammatory responses to bacterial agonists such as lipopolysaccharide (LPS). Furthermore, we show that distal macrophages from 4T1 tumor-bearing mice exhibited increased pro-inflammatory cytokine responses to LPS. The propensity of tumor-conditioned macrophages to strongly respond to bacterial agonists may have profound implications on breast cancer progression and innate microbial host defense in breast cancer patients.

## Results

### Increased production of LPS-induced pro-inflammatory cytokines by tumor-conditioned BMDMs

**Fig 1:**
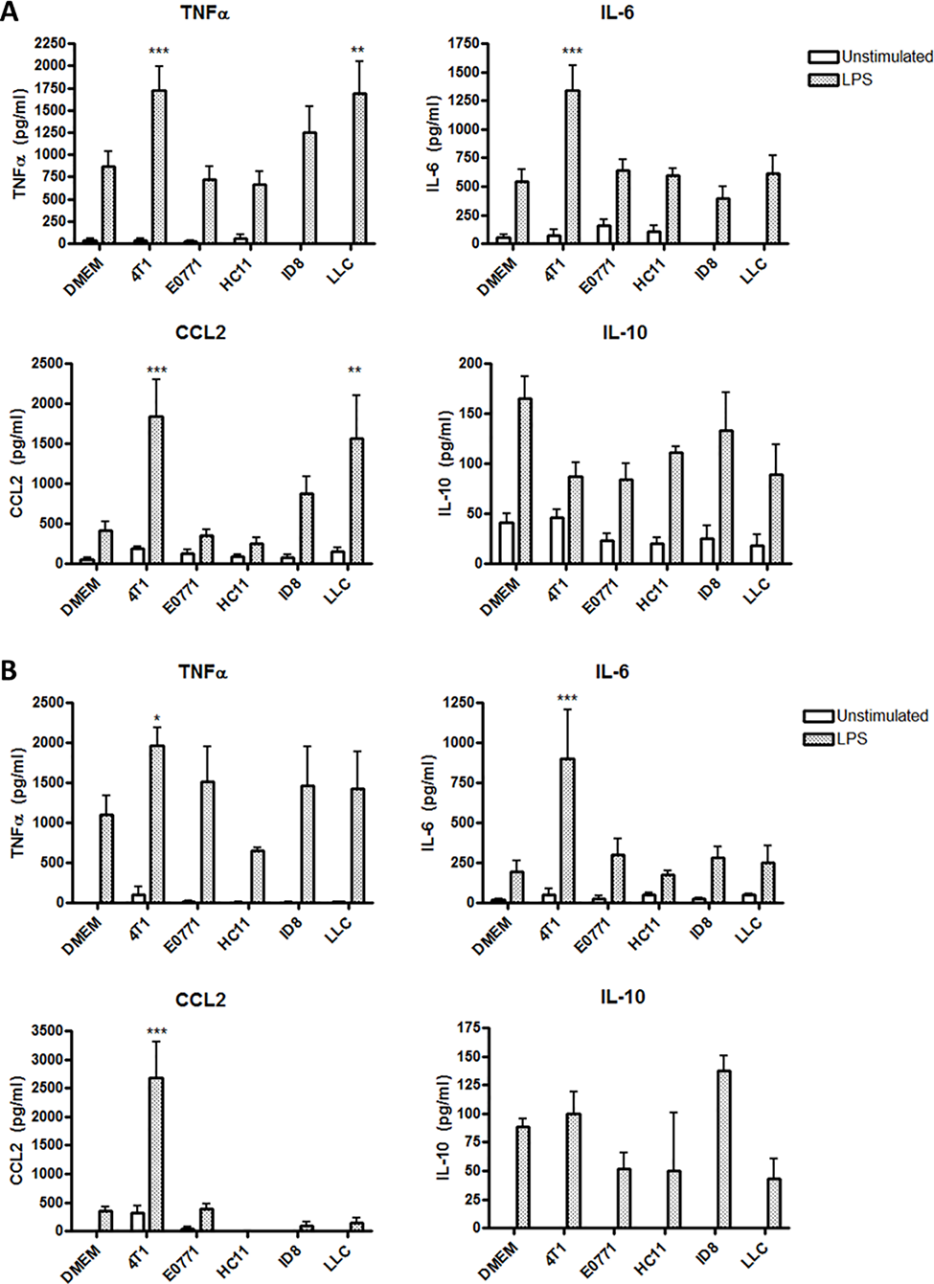
4T1- and LLC-conditioned macrophages exhibit increased pro-inflammatory cytokine production in response to LPS. (A) C57BL/6 or (B) BALB/c BMDMs were cultured in DMEM alone or in the presence of culture medium conditioned by 4T1 mammary carcinoma, E0771 mammary carcinoma, ID8 ovarian carcinoma, or LLC cells for 24 h. Medium conditioned by HC11 normal mouse epithelial cells was used as a negative control. BMDMs were then washed with PBS and stimulated with 100 ng/ml LPS for 4 h, then culture supernatants were collected and protein levels of cytokines TNFα, IL-6, CCL2, and IL-10 were determined by ELISA. Results are shown as the mean (± SEM) of at least 5 independent experiments. Statistical analyses were done by ANOVA, followed by Tukey’s multiple comparison tests; * p < 0.05, ** p < 0.01, *** p < 0.001.

To assess the inflammatory phenotype of tumor-conditioned macrophages, BMDMs were exposed to tumor-conditioned media for 24 h and then stimulated with 100 ng/ml LPS from *Escherichia coli* for 4 h. As expected, LPS stimulation of non-conditioned BMDMs from C57BL/6 or BALB/c mice resulted in the production of pro-inflammatory cytokines tumor necrosis factor α (TNFα) and IL-6, macrophage chemokine CCL2, and anti-inflammatory cytokine IL-10 (Fig 1A and 1B). Surprisingly, C57BL/6 BMDMs cultured with 4T1 mouse mammary carcinoma-conditioned medium exhibited a 2–3 fold increase in the production of TNFα, IL-6, and CCL2 in the presence of LPS (Fig 1A). Lewis lung carcinoma (LLC)-conditioned BMDMs also showed a significant increase in TNFα and CCL2 levels in response to LPS. ID8 ovarian carcinoma-conditioned BMDMs demonstrated a modest increase in TNFα and CCL2 following LPS stimulation, although this effect was not statistically significant. In contrast, none of these tumor-conditioned media affected LPS-stimulated IL-10 production by BMDMs when compared to non-conditioned BMDMs. Neither E0771 mouse mammary carcinoma-conditioned medium nor control medium conditioned with HC11 mouse mammary epithelial cells affected the LPS response of BMDMs. Similar trends were observed in macrophages from BALB/c mice; 4T1-conditioned Balb/c BMDMs showed a striking increase in TNFα, IL-6, and CCL2 production in response to LPS, while IL-10 levels remained unaffected (Fig 1B), demonstrating that this effect was not mouse strain-specific. TNFα and IL-6 were not detected in the tumor-conditioned media nor in the supernatants of conditioned BMDMs, prior to LPS stimulation, indicating that the tumor-conditioning step itself did not elicit an inflammatory response (data not shown). These results indicate that secreted factors of 4T1 murine mammary carcinomas and LLC can potentiate macrophage inflammatory cytokine production in response to LPS. Furthermore, this increased sensitivity is selective since TNFα, IL-6, and CCL2 production was increased while anti-inflammatory cytokine IL-10 production was unaffected. Subsequent experiments employed 4T1-conditioned C56BL/6 BMDMs because the effect was most striking in these cells.

### Chemokine production and cell recruitment by 4T1-conditioned BMDMs

**Fig 2:**
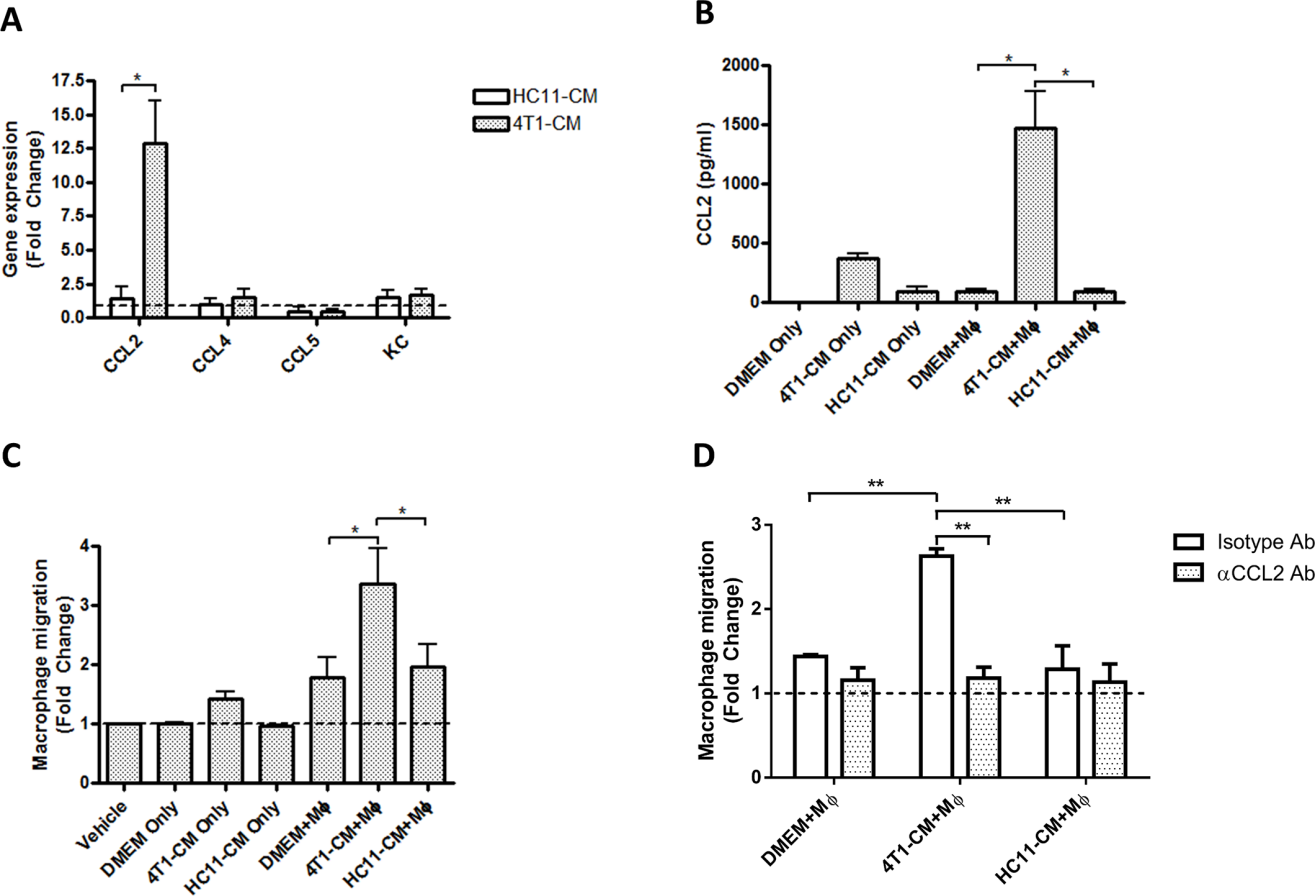
Macrophages exposed to 4T1-conditioned culture medium demonstrate increased CCL2 production and promote macrophage recruitment. (A) Gene expression of monocyte/macrophage chemokines by BMDMs treated for 24 h with 4T1- or HC11-conditioned medium (CM). Expression levels are displayed as the fold change relative to gene expression of BMDMs treated with DMEM alone. (B) CCL2 protein production by BMDMs (Mϕ) treated for 24h with 4T1- or HC11-conditioned medium as measured by ELISA. (C) Macrophage chemotaxis towards the culture supernatants of 4T1- or HC11-conditioned BMDMs (Mϕ). (D) Macrophage chemotaxis towards macrophage culture supernatants pre-treated with 10 μg/ml of a CCL2-neutralizing or an isotype Ab for 1h. Results are shown as the mean (± SEM) of at least 3 independent experiments. Statistical analyses were done by ANOVA, followed by Tukey’s multiple comparison tests; * p < 0.05.

Because increased expression of pro-inflammatory cytokines by tumor-conditioned macrophages in response to LPS was unexpected, we confirmed the phenotype of our 4T1-conditioned BMDMs with behaviors that are well established in the literature. One hallmark of TAMs is their elevated production of monocyte/macrophage chemokines, which results in enhanced macrophage recruitment to the tumor site [6,16]. For example, co-cultures of 4T1 cells and murine macrophages exhibit greatly increased mRNA expression and secretion of monocyte chemoattractant CCL2[15]. We therefore isolated RNA from 4T1-conditioned BMDMs and determined mRNA expression levels of a number of monocyte chemokines by real-time quantitative PCR. BMDMs treated with medium conditioned by HC11 mammary epithelial cells were used as non-tumor controls. HC11-conditioned BMDMs had comparable mRNA levels of CCL2, CCL4, CCL5, and neutrophil chemoattractant KC to non-conditioned BMDMs (Fig 2A). 4T1-conditioned BMDMs did not exhibit increased mRNA expression of CCL4, CCL5, or KC, but showed a strong 12-fold increase in CCL2 expression. This finding was confirmed at the protein level by assessing CCL2 levels in culture supernatants of 4T1-conditioned BMDMs, which showed greater production of CCL2 protein compared to non-conditioned or HC11-conditioned BMDMs (Fig 2B). 4T1-conditioned medium alone had modest levels of CCL2, which is in line with reports that 4T1 cells secrete CCL2[15,17]; however, CCL2 levels were far below the levels produced by 4T1-conditioned BMDMs.

To determine whether 4T1-conditioned macrophages could promote the subsequent recruitment of new macrophages, supernatants from cultures of 4T1-conditioned BMDMs were used as a chemoattractants for freshly isolated BMDMs. Control supernatants from non-conditioned and HC11-conditioned BMDMs caused a 1.8-fold increase in BMDM migration through a porous membrane contained in a chemotaxis chamber, indicating baseline production of chemoattractants by untreated BMDMs (Fig 2C). Supernatants from 4T1-conditioned BMDMs caused a greater than 3-fold increase in BMDM migration. BMDM migration towards tumor-conditioned medium alone was determined to control for the presence of pre-existing chemokines in the medium. Non-conditioned and HC11-conditioned culture medium did not promote BMDM migration while 4T1-conditioned medium caused a slight 1.4-fold increase in BMDM migration. These trends are in line with the observed CCL2 mRNA expression and protein secretion; however, this does not preclude an effect by other chemokines that may be produced by tumor-conditioned macrophages. To confirm the role of chemokine CCL2, BMDM culture supernatants were incubated with a CCL2-neutralizing antibody or an isotype-matched antibody for 1h prior to their use in chemotaxis assays. CCL2 neutralization fully inhibited cell migration towards the supernatants of 4T1-conditioned BMDMs (Fig 2D), firmly establishing the role of this chemokine in macrophage recruitment. Taken together, these results show that 4T1-conditioned BMDMs show enhanced production of macrophage chemoattractants.

### Enhanced LPS-sensitivity of 4T1-conditioned BMDMs is transient

**Fig 3:**
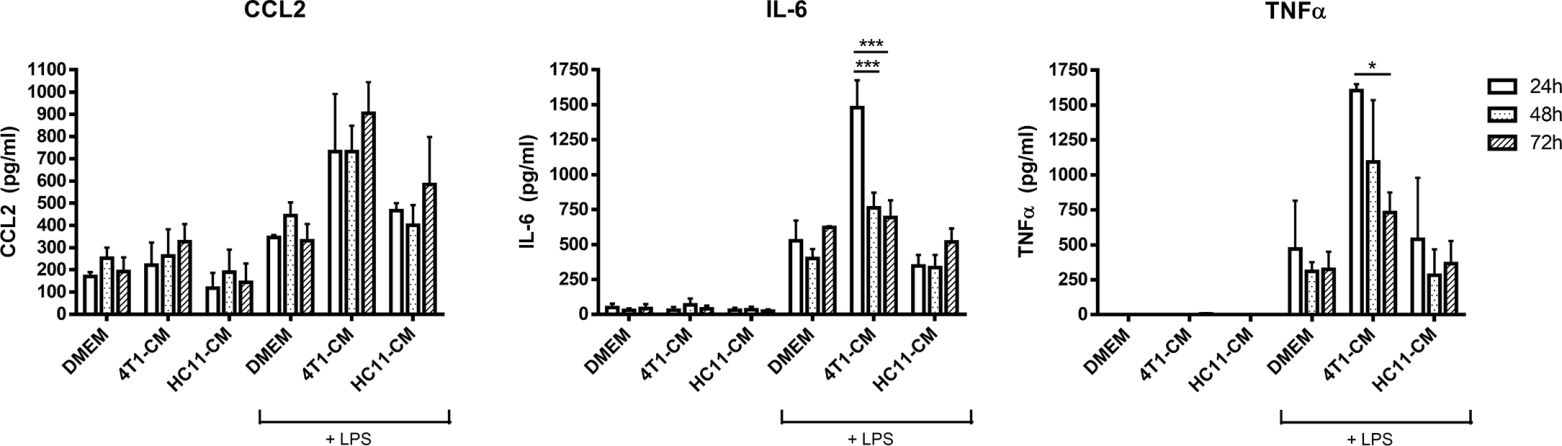
Increased sensitivity of 4T1-conditioned macrophages to LPS is transient. BMDMs were treated with 4T1- or HC11-conditioned medium (CM) for the indicated times. Following treatment, BMDMs were then washed with PBS and stimulated with 100 ng/ml LPS for 4 h. Culture supernatants were collected and protein levels of cytokines CCL2, TNFα, and IL-6 were determined by ELISA. Results are the mean (± SEM) of 3 independent experiments. Statistical analyses were done by ANOVA, followed by Tukey’s multiple comparison tests; * p < 0.05.

We next performed a time-course conditioning experiment to determine whether increased sensitivity of 4T1-conditioned BMDMs to LPS was stable. BMDMs were treated with 4T1-conditioned medium for 24 h, 48 h, or 72 h, then washed and stimulated for 4 h with 100 ng/ml of LPS. 4T1-conditioning alone induced the moderate production of CCL2 by BMDMs yet had no effect on IL-6 or TNFα production (Fig 3). After 24 h of conditioning, 4T1-conditioned BMDMs showed greatly elevated CCL2, IL-6, and TNFα production in response to LPS (Fig 3), as was seen previously. However, this elevated cytokine production was reduced over time, as evidenced by the lower levels of IL-6 and TNFα at 48h and a near-elimination of the response by 72 h. Interestingly, enhanced LPS-induced CCL2 production by 4T1-conditioned macrophages was evident up to 72 h of tumor-conditioning. 4T1-secreted factors therefore transiently potentiated macrophage pro-inflammatory cytokine responses to LPS. Production of CCL2, TNFα, and IL-6 by HC11-conditioned BMDMs in response to LPS was unchanged, regardless of conditioning time.

### Increased production of pro-inflammatory cytokines by 4T1-conditioned BMDMs stimulated with flagellin, peptidoglycan and CpG ODN

**Fig 4:**
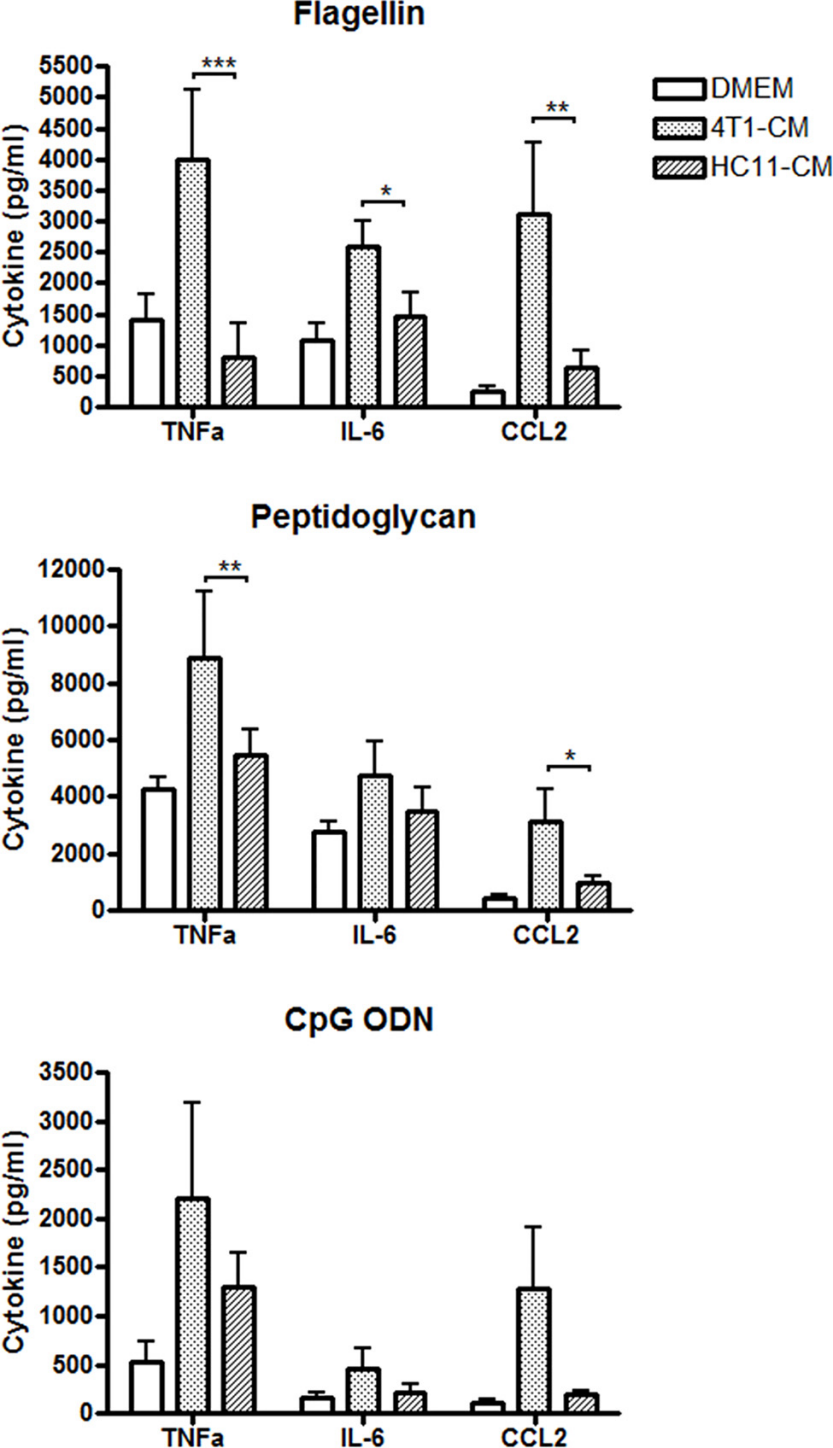
4T1-conditioned macrophages show increased inflammatory cytokine production in response to bacterial agonists. BMDMs were treated with 4T1- or HC11-conditioned medium (CM) for 24 h. BMDMs were then washed with PBS and stimulated for 24 h with CpG ODN (5 μg/ml), flagellin (5 μg/ml), or peptidoglycan (10 μg/ml). Culture supernatants were collected and protein levels of cytokines TNFα, IL-6, and CCL2 were determined by ELISA. Results are shown as the mean (± SEM) of at least 5 independent experiments. Statistical analyses were done by ANOVA, followed by Tukey’s multiple comparison tests; * p < 0.05, ** p < 0.01, *** p < 0.001.

We next determined whether 4T1-conditioned BMDMs had increased sensitivity to other inflammatory agonists or whether the effect was specific to LPS. BMDMs were treated with tumor-conditioned medium for 24 h, washed, and exposed to a number of bacterial agonists for 24 h. As before, BMDMs conditioned with 4T1 or HC11 culture medium in the absence of agonists showed no change in cytokine levels when compared with non-conditioned BMDMs (data not shown). In contrast, 4T1-conditioned BMDMs exhibited increased cytokine production in response to *Salmonella typhimurium* flagellin, with a 5-fold, 2-fold, and 6-fold increase in TNFα, IL-6, and CCL2 levels, respectively, in comparison to control HC11-conditioned BMDMs (Fig 4). A similar effect was seen when 4T1-conditioned BMDMs were stimulated with *E*. *coli* peptidoglycan, resulting in TNFα and CCL2 production to levels that were 2–3 times greater than the amount produced by HC11-conditioned BMDMs. Interestingly, IL-6 production by 4T1-conditioned BMDMs was only elevated by 25% compared to HC11-conditioned BMDMs, an effect which was not statistically significant. Treatment of 4T1-conditioned BMDMs with CpG ODN resulted in a 2-fold increase in TNFα and IL-6 production, and a 5-fold increase in CCL2 levels when compared with HC11-conditioned BMDMs, although this effect was not statistically significant. Overall, these experiments show that 4T1-priming of macrophage inflammatory responses is not limited to LPS alone, but can also be mediated by other bacterial agonists.

### Enhanced phagocytosis and production of reactive nitrogen species by 4T1-conditioned BMDMs

**Fig 5:**
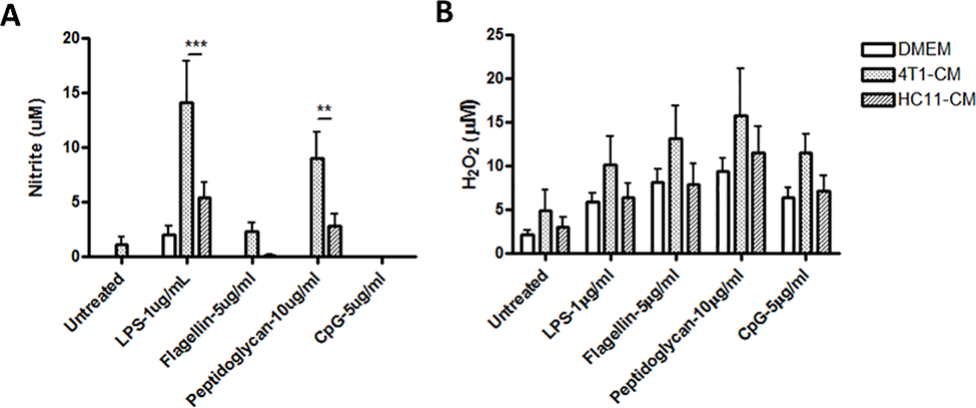
4T1-conditioned macrophages demonstrate increased nitrite production in response to LPS. BMDMs were treated with 4T1- or HC11-conditioned medium for 24 h. (A) To assess nitrite production, BMDMs were then washed with PBS followed by stimulation with LPS (100 ng/ml), flagellin (5 μg/ml), peptidoglycan (10 μg/ml), or CpG ODN (5 μg/ml) for another 24 h. Culture supernatants were collected and the nitrite production was assessed by the addition of Griess reagent for 15 min. Absorbance (570 nm) of the culture supernatants was then measured by a spectrophotometer. (B) To measure the production of reactive oxygen species, tumor-conditioned BMDMs were stimulated with bacterial TLR agonists for 24 h in the presence of Amplex Red reagent and horseradish peroxidase. Absorbance (450 nm) of the cultures was then measured by a spectrophotometer. Results are shown as the mean (± SEM) of at least 4 independent experiments. Statistical analyses were done by ANOVA, followed by Tukey’s multiple comparison tests; ** p < 0.01, *** p < 0.001.

Since 4T1-conditioning augmented macrophage-mediated inflammatory responses to several different bacterial agonists, we focused on the implications that this effect may have on innate inflammatory functions and host defense. To address this, we first investigated the effects of 4T1 factors on macrophage production of reactive nitrogen and oxygen intermediates. 4T1-conditioned BMDMs were stimulated with LPS, flagellin, peptidoglycan, or CpG ODN for 24 h prior to the measurement of reactive nitrogen species in culture supernatants. In control HC11-conditioned BMDMs, only LPS and peptidoglycan elicited detectable nitrite production after 24 h (Fig 5A). However, nearly a 3-fold increase in nitrite levels was detected in 4T1-conditioned BMDMs stimulated with LPS and peptidoglycan. Similar experiments were performed to assess the production of reactive oxygen intermediates by tumor-conditioned macrophages. There was a trend towards elevated hydrogen peroxide production by 4T1-conditioned BMDMs in response to stimulation with bacterial agonists tested (Fig 5B); however, this effect was not statistically significant. Culture supernatants of non-conditioned and HC11-conditioned BMDMs showed comparable hydrogen peroxide production after 24 h of agonist stimulation.

**Fig 6:**
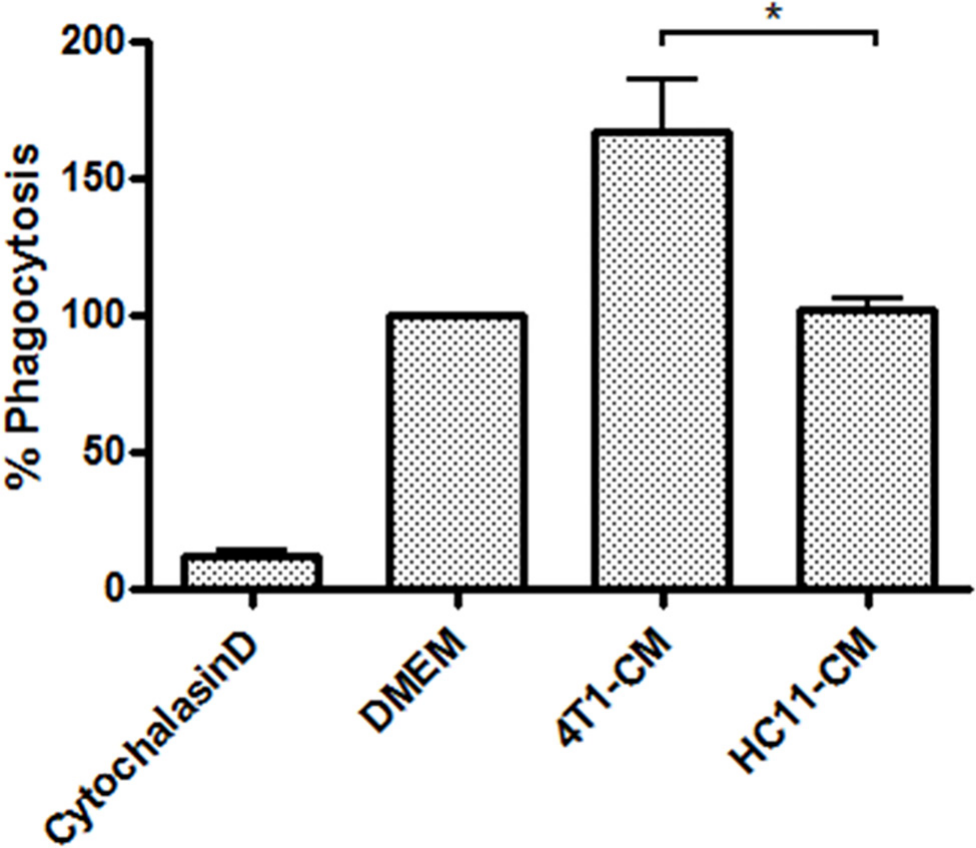
4T1-conditioned macrophages exhibit increased phagocytosis of *E*. *coli* bioparticles. BMDMs were treated for 24 h with 4T1- or HC11-conditioned medium (CM). BMDMs were then washed with PBS and incubated for 2 h with *E*. *coli* bioparticles, which fluoresce only under acidic conditions present in endosomes. *E*. *coli* phagocytosis was determined by measuring BMDM fluorescence by flow cytometry. As a negative control, BMDMs were pre-treated for 1 h with actin polymerization inhibitor cytochalasin D (10 μM) prior to the addition of *E*. *coli* bioparticles. Phagocytic activity was normalized to the uptake of *E*. *coli* bioparticles by DMDMs treated with DMEM alone. Results are shown as the mean (± SEM) of at least 4 independent experiments. Statistical analyses were done by ANOVA, followed by Tukey’s multiple comparison tests; * p < 0.05.

Phagocytosis of microorganisms is one of the hallmark functions of macrophage-mediated host defense. Considering the enhancing effect of 4T1-conditioning on macrophage-mediated cytokine production and nitrite secretion, we next investigated the effect of tumor conditioning on phagocytosis. BMDMs were treated with 4T1- or HC11-conditioned culture medium for 24 h prior to incubation with pHrodo *E*. *coli* bioparticles, which fluoresce only under acidic conditions such as are found in late endosomes. After 2 h of incubation with bioparticles, HC11-conditioned control BMDMs showed comparable ingestion of bioparticles to non-conditioned BMDMs, whereas 4T1-conditioned BMDMs demonstrated a greater than 50% increase in phagocytic activity (Fig 6). These experiments show that 4T1-mediated enhancement of macrophage sensitivity to bacterial agonists involves other aspects of innate host defense and is not limited to enhanced pro-inflammatory cytokine production.

### Elevated expression of F4/80 on the surface of 4T1-conditioned BMDMs

**Fig 7:**
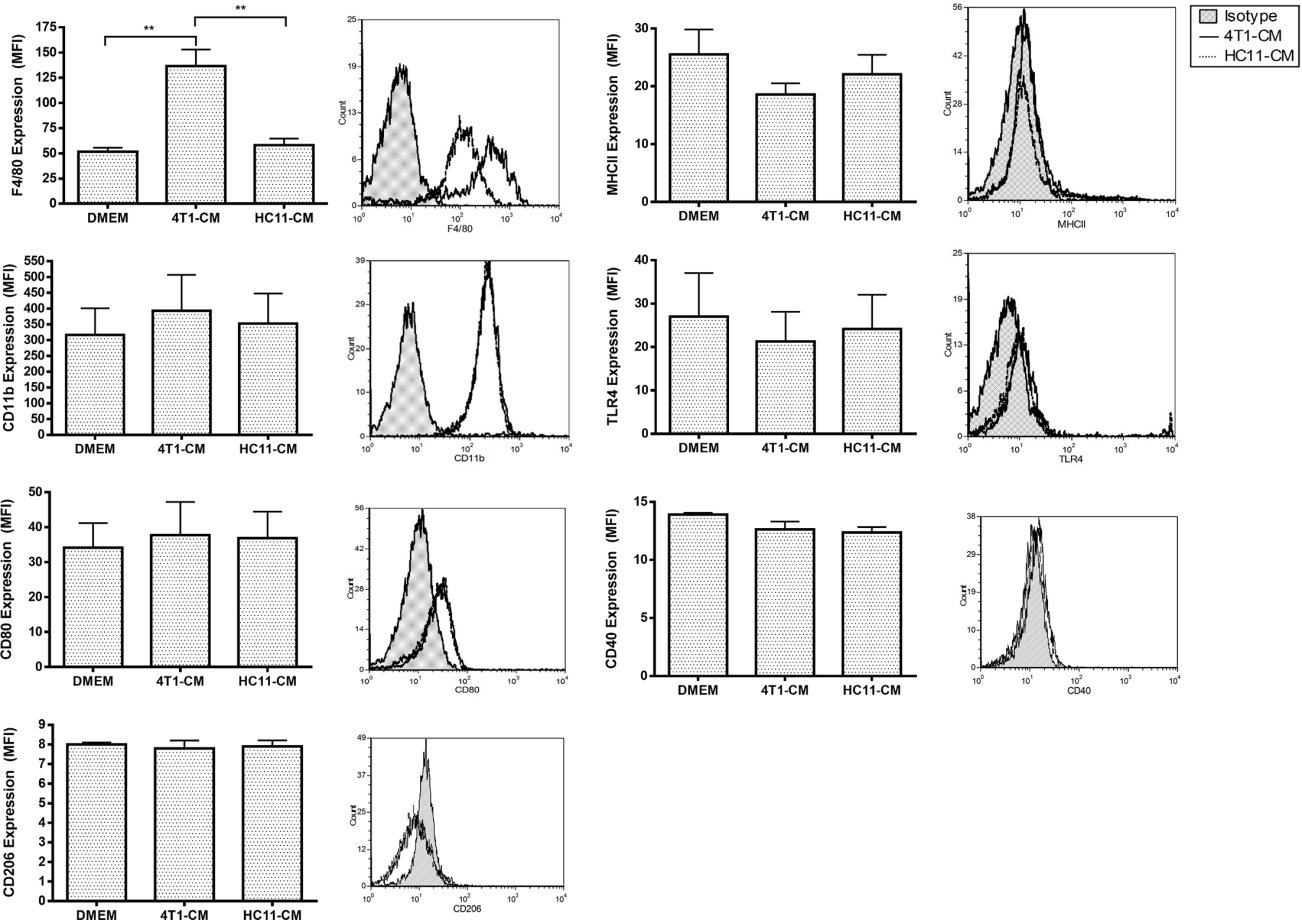
Increased F4/80 expression by 4T1-conditioned macrophages. BMDMs were treated for 24 h with 4T1- or HC11-conditioned medium (CM). BMDMs were stained with fluorochrome-labeled antibodies against macrophage markers F4/80, CD11b, CD80, and MHC II, as well as receptors CD40 and CD206. Cell fluorescence was measured via flow cytometry. Protein surface expression of tumor-conditioned BMDMs is expressed as the geometric mean fluorescence intensity. Results are the mean (± SEM) of at least 4 independent experiments. Statistical analyses were done by ANOVA, followed by Tukey’s multiple comparison tests; ** p < 0.01.

The profound effects of 4T1-conditioning on macrophage function lead us to investigate cell surface expression of macrophage markers for clues regarding potential macrophage reprogramming. 4T1-conditioned BMDMs demonstrated no change in CD11b expression compared to non-conditioned and HC11-conditioned control BMDMs, but showed nearly a 3-fold increase in F4/80 expression (Fig 7). There was no significant change in the levels of co-stimulatory receptor CD80, antigen-presenting MHC class II molecules, or TLR4 surface expression by 4T1-conditioned BMDMs compared to control BMDMs. To determine whether the effects of 4T1 tumor-conditioning are due to M1/M2 macrophage polarization, CD40 and CD206 surface expression was also investigated; however, no changes were observed in the expression of these receptors by 4T1-conditioned BMDMs.

### Actin polymerization is necessary for the increased activity of 4T1-conditioned BMDMs

**Fig 8:**
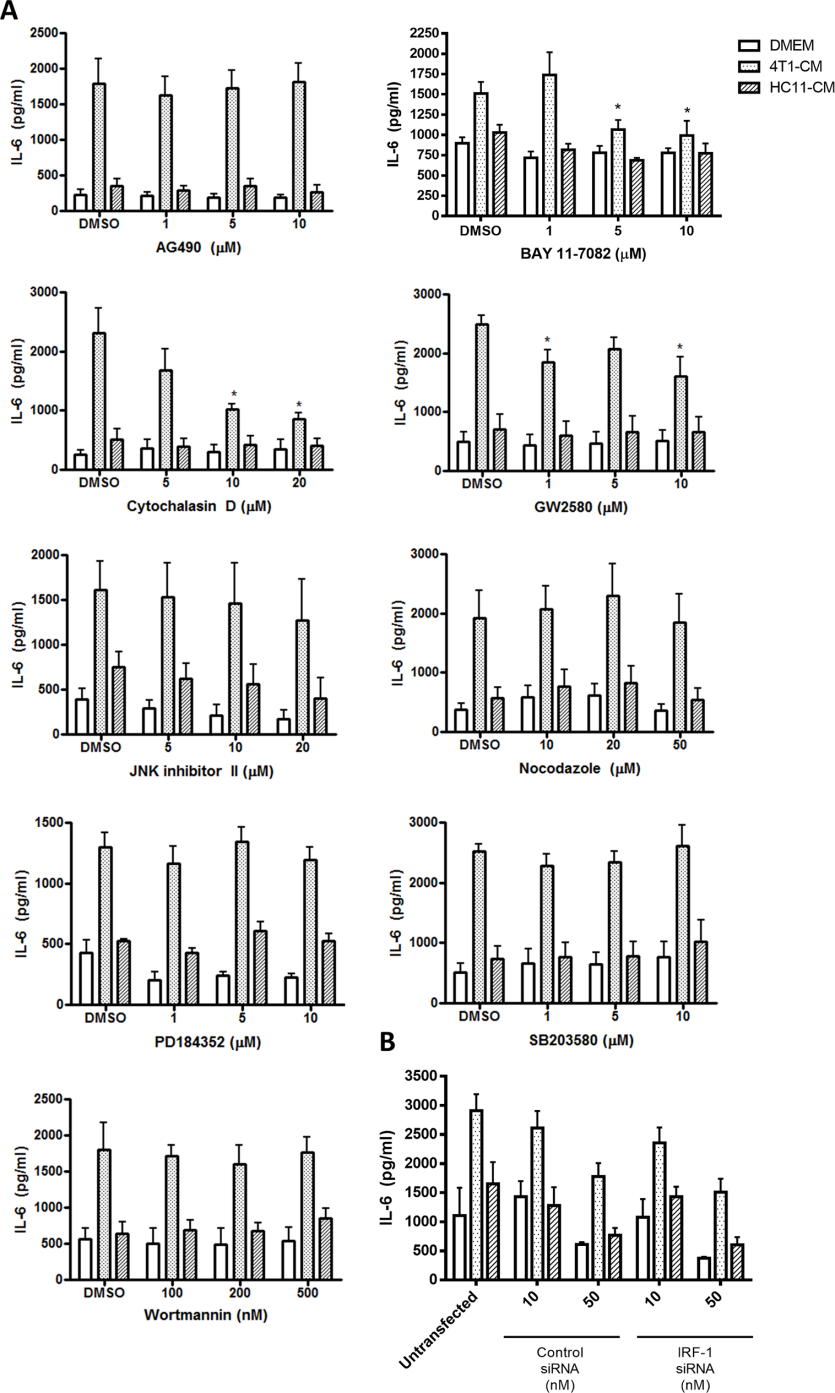
The increased sensitivity of 4T1-conditioned macrophages to LPS is dependent on actin polymerization. (A) BMDMs were pre-treated for 1 h with the indicated concentrations of AG490, BAY 11–7082, cytochalasin D, GW2580, JNK inhibitor II, nocodazole, PD184352, SB203580, wortmannin, or the DMSO vehicle prior to a 24 h treatment with 4T1- or HC11-conditioned medium (CM). (B) BMDMs were transfected with IRF-1 or control siRNA for 24 h prior to a 24 h treatment with 4T1- or HC11-CM. BMDMs were then washed with PBS and stimulated with 100 ng/ml LPS for 4 h. Culture supernatants were collected and assessed for the levels of IL-6 by ELISA. Results are shown as the mean (± SEM) IL-6 levels of at least 4 independent experiments. Statistical analyses were done by ANOVA, followed by Tukey’s multiple comparison tests; * p < 0.05.

To begin to elucidate the mechanism through which 4T1-secreted factors primed the macrophage inflammatory response, we used pharmacologic inhibitors of certain key cellular signaling pathways during the tumor-conditioning step. BMDMs were pre-treated for 1 h with chemical inhibitors of specific pathways prior to the addition of tumor-conditioned medium. BMDMs were then maintained in tumor-conditioned medium and pathway inhibitors for 24 h, washed with PBS to remove the inhibitor and conditioned medium, and stimulated with LPS for 4 h. As before, compared to HC11-conditioned control BMDMs, 4T1-conditioned BMDMs that were cultured with the DMSO vehicle showed elevated IL-6 production in response to LPS (Fig 8A). Since increased production of IFNγ was reported in co-cultures of 4T1 cells and murine macrophages[15], we investigated whether IFNγ receptor signaling was responsible for the transient increase in macrophage sensitivity to bacterial agonists. However, pre-treatment with JAK2 inhibitor AG490 did not affect IL-6 production by 4T1-conditioned macrophages, excluding a role of IFNγ and cytokine-mediated JAK-STAT signalling. Likewise, siRNA-mediated knockdown of interferon regulatory factor (IRF)-1 had no effect on LPS-induced IL-6 production by 4T1-conditioned BMDMs, further precluding a role of interferons in this phenomenon (Fig 8B). Furthermore, pre-treatment with phosphatidylinositol 3-kinase pathway inhibitor wortmannin, p38 mitogen-activated protein kinase pathway inhibitor SB203580, MEK inhibitor PD184352, and JNK pathway inhibitor II, did not interfere with increased IL-6 production by 4T1-conditioned BMDMs that were stimulated with LPS (Fig 8A). In contrast, pre-treatment with NFκB inhibitor BAY 11–7082 abrogated LPS-induced IL-6 production in 4T1-conditioned BMDMs, suggesting a role for the NFκB pathway in the tumor-conditioning of BMDMs. Since 4T1 mammary carcinoma cells are known to secrete macrophage colony stimulating factor (M-CSF), we speculated that this cytokine could be responsible for the increased inflammatory phenotype of our conditioned macrophages. Pre-treatment with 10 μM of GW2580, an inhibitor of M-CSF receptor (CSF-1R), reduced IL-6 production of 4T1-conditioned BMDMs by nearly 40%, indicating a partial role of this cytokine in the increased sensitivity of 4T1-conditioned macrophages to LPS. Inhibitors of the cytoskeletal system were used to determine whether macrophage uptake of 4T1-secreted mediators was required for the observed effects. Inhibition of actin polymerization with cytochalasin D during the conditioning step resulted in a dose-dependent inhibition of IL-6 production by 4T1-conditioned BMDMs that were stimulated with LPS, suggesting that actin-mediated internalization of 4T1-secreted mediators by macrophages is likely required for the observed increase in inflammatory responses. In contrast, the microtubule polymerization inhibitor nocodazole had no effect on the response of 4T1-conditioned BMDMs to LPS, suggesting that endosomal trafficking played little role in the conditioning process. None of the pharmacologic inhibitors affected LPS-stimulated IL-6 production by HC11- and non-conditioned control BMDMs.

### 4T1-secreted vesicles are involved in priming macrophage inflammatory responses

**Fig 9:**
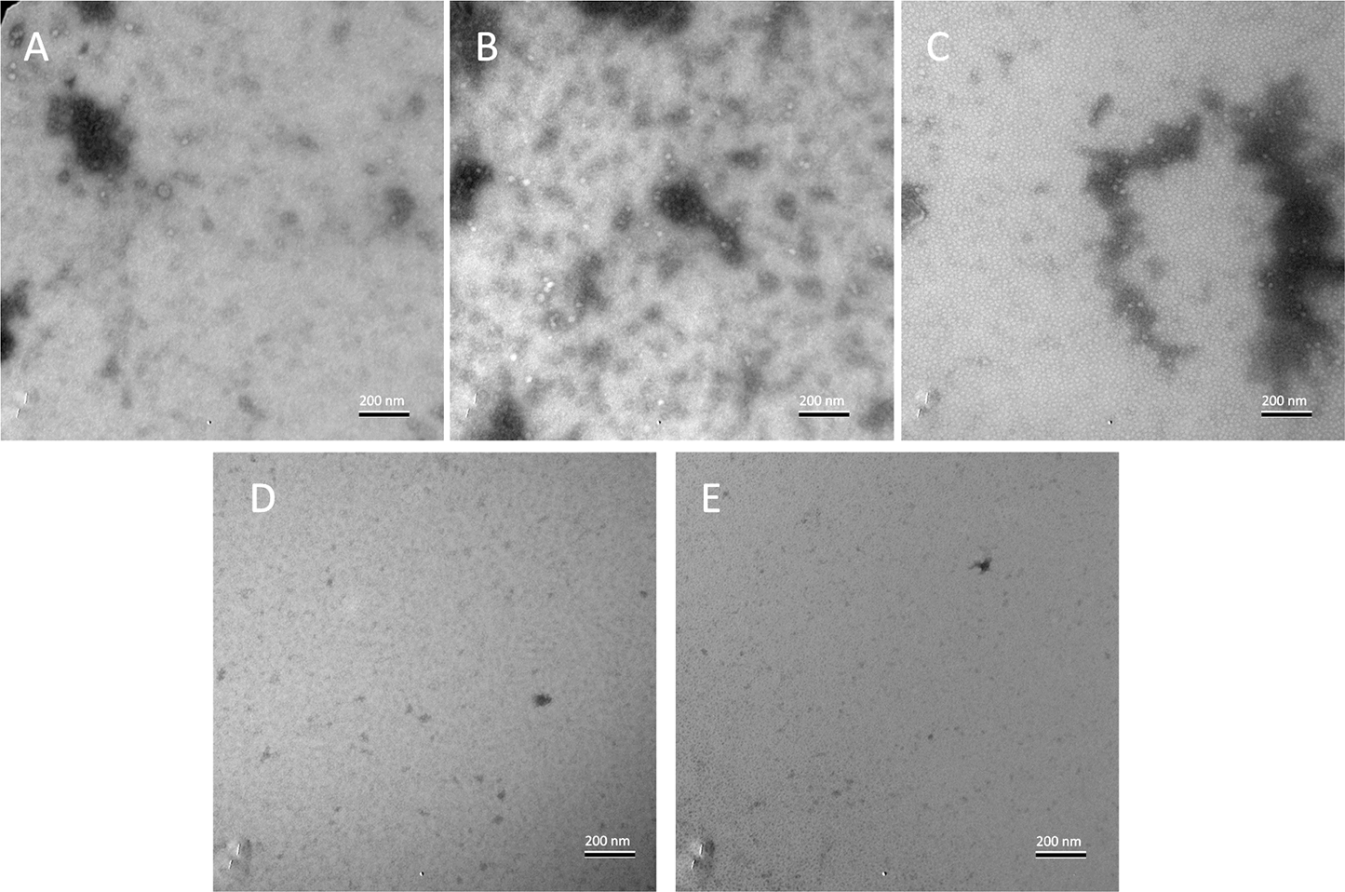
4T1- and HC11-conditioned media contain extracellular vesicles. Conditioned culture media were absorbed onto carbon-coated grids, negatively stained with 1% uranyl acetate, and visualized by electron microscopy. The displayed images are representative images of (A) fresh medium, (B) 4T1-conditioned medium, (C) HC11-conditioned medium, (D) sonicated 4T1-conditioned medium, and (E) sonicated HC11-conditioned medium.

**Fig 10:**
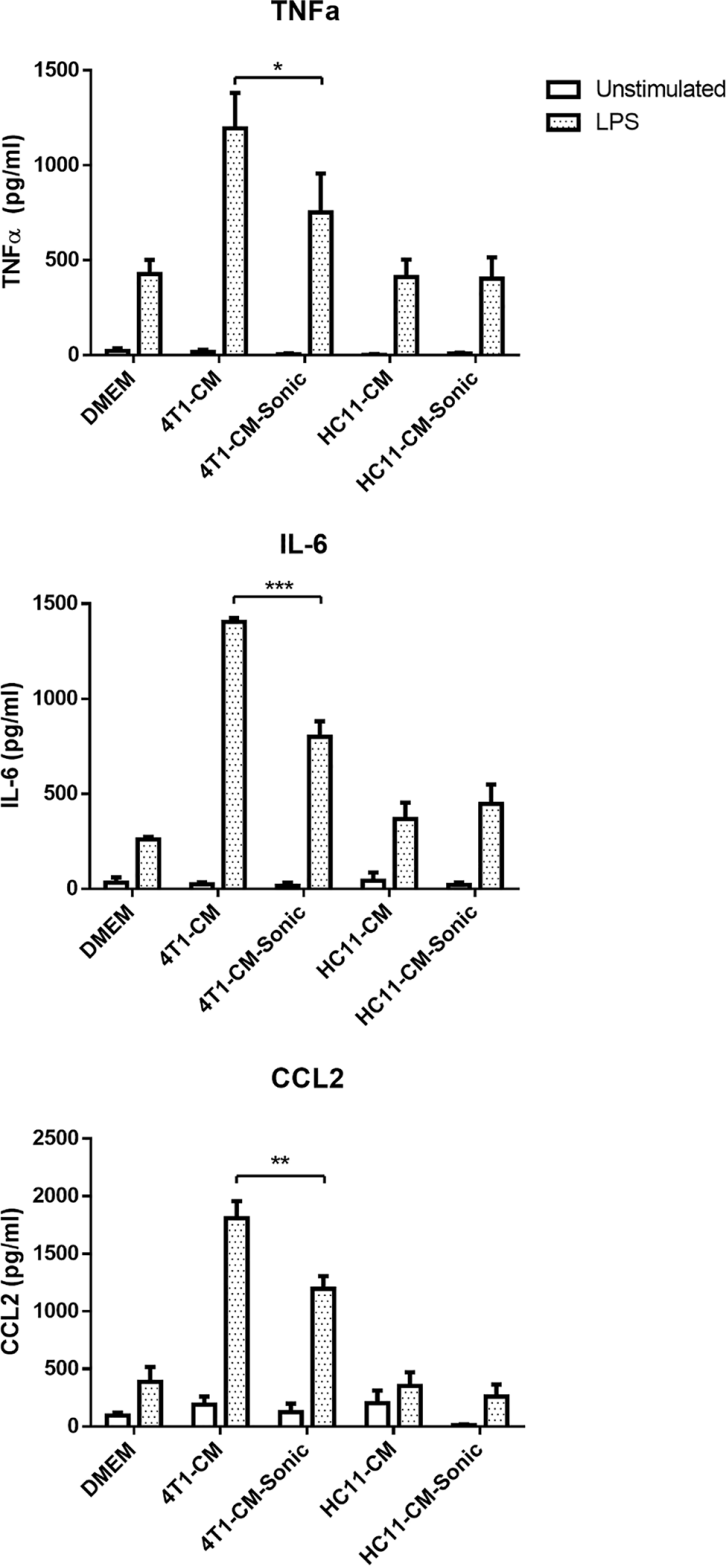
Disruption of extracellular vesicle integrity diminishes 4T1-mediated priming of macrophage inflammatory responses. BMDMs were treated for 24 h with sonicated 4T1- or HC11-conditioned medium (CM). BMDMs were then washed with PBS and stimulated with 100 ng/ml LPS for 4 h. Culture supernatants were collected and assessed for the levels of cytokines by ELISA. Results are shown as the mean (± SEM) cytokine levels of at least 3 independent experiments. Statistical analyses were done by ANOVA, followed by Tukey’s multiple comparison tests; * p < 0.05, ** p < 0.01, *** p < 0.001.

Since 4T1-mediated priming of macrophage inflammatory responses involved actin-mediated uptake of tumor-secreted factors, we investigated the possible involvement of tumor-secreted vesicles such as exosomes, which play a major role in exerting distal effects on numerous cell types, including macrophages[18]. To determine whether extracellular vesicles were being secreted by 4T1 cells, electron microscopy was performed on tumor-conditioned culture medium. In fresh medium, extracellular vesicles approximately 40 nm in diameter were detected, likely stemming from serum-sourced exosomes (Fig 9A). In comparison, both 4T1-conditioned medium and HC11-conditioned control medium showed greatly increased levels of extracellular vesicles that were approximately 30–40 nm in diameter (Fig 9B and 9C). This finding was in line with the observation that normal epithelial cells and tumor cells are sources of exosomes [18], and confirms that 4T1-secreted vesicles were present in the carcinoma-conditioned medium. To determine whether vesicles secreted by 4T1 mammary carcinoma cells were responsible for regulating macrophage responses, 4T1-conditioned medium, as well as HC11-conditioned control medium, were cleared of vesicles by sonication. Inspection by electron microscopy confirmed that sonication of 4T1- or HC11-conditioned medium completely eliminated extracellular vesicles (Fig 9D and 9E). Vesicle-cleared culture media were then used to prime BMDMs for 24 h prior to stimulation with LPS. After 4h of LPS treatment, 4T1-conditioned BMDMs showed enhanced production of TNFα, IL-6, and CCL2 compared to non-conditioned or HC11-conditioned control BMDMs, as previously observed, whereas sonication of 4T1-conditioned medium prior to macrophage treatment resulted in a partial inhibition of LPS-induced pro-inflammatory cytokine release (Fig 10). In contrast, sonication of HC11-conditioned media had no effect on BMDM cytokine production in response to LPS. These results, in conjunction with the inhibitory effect of cytochalasin D, suggest that actin-mediated uptake of 4T1-secreted vesicles by macrophages contributed to enhanced inflammatory responses.

### Peritoneal macrophages of 4T1 tumor-bearing mice are more sensitive to LPS stimulation

**Fig 11:**
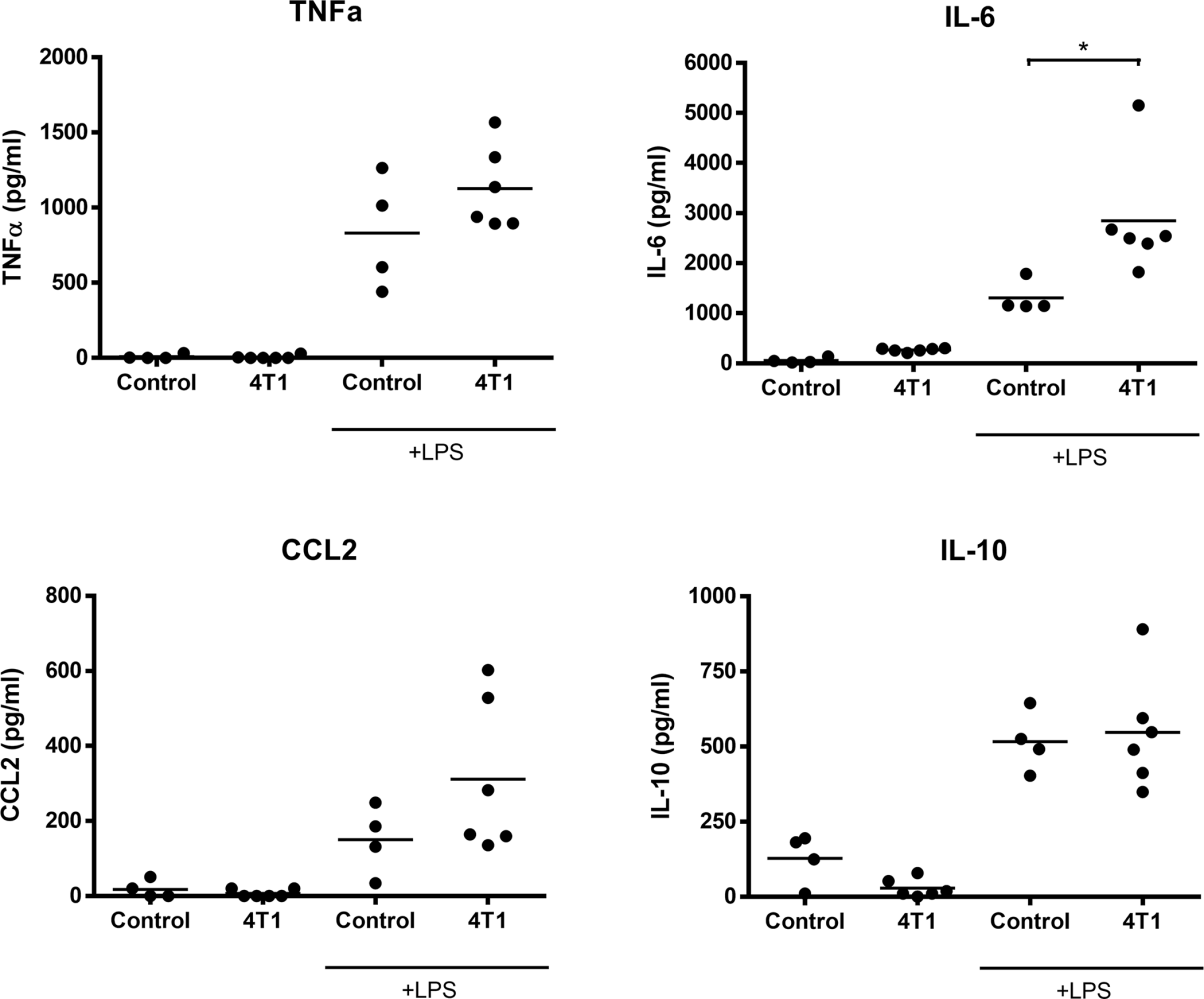
Peritoneal macrophages from 4T1 tumor-bearing mice demonstrate enhanced sensitivity to LPS. Peritoneal macrophages from control (n = 4) or 4T1-bearing mice (n = 6) were stimulated with 100 ng/ml LPS for 4 h. Culture supernatants were collected and assessed for the levels of cytokines by ELISA. Statistical analyses were done by ANOVA, followed by Tukey’s multiple comparison tests; * p < 0.05.

To determine whether 4T1 mammary carcinoma cells can influence the inflammatory behavior of macrophages distal to the primary tumor site, peritoneal macrophages were isolated from non-tumor-bearing and 4T1 tumor-bearing BALB/c mice and stimulated with LPS for 4h prior to measuring cytokine production. Similar to the effects seen in BMDMs, peritoneal macrophages from 4T1 tumor-bearing mice exhibited significantly strengthened production of IL-6 and moderately increased production of CCL2 and TNFα in response to LPS when compared to macrophages from control mice (Fig 11). In contrast, the presence or absence of 4T1 tumors had no effect on the LPS-induced production of IL-10 by peritoneal macrophages. These findings demonstrate that 4T1 tumors can exert distal effects on macrophage behavior towards bacterial agonists.

## Discussion

The interaction between inflammation and the cancer microenvironment has profound effects on tumor progression and, thus, patient mortality. Carcinomas transform their local tissue environment, often subverting the inflammatory response to regulate tissue remodeling, promoting angiogenesis and cancer cell motility, which contributes to metastasis[2]. Furthermore, tumor cells frequently skew the immune response to evade and impair cell-mediated anti-cancer immunity[1]. While cancers often impact on host immunity, the reverse is also true since inflammatory conditions often precede the development of malignancies[11,19]. At the junction of the complex interactions between inflammation and cancer are cells of the immune system, including TAMs that are often the key mediators through which cancers alter their local environment. While a large number of studies have focused on TAM behavior in the context of tissue remodeling, recruitment of suppressor cells, and skewing of adaptive immunity, little attention has been given to the possible impact of tumors on macrophage function in innate host defense.

In this study, we describe the effect of 4T1 murine mammary carcinomas on inflammatory responses by macrophages. Exposure to 4T1 mediators strongly induced BMDM production of CCL2, which was consistent with a report by Cho *et al*. [15], whereas in our study production of TNFα and IL-6 remained unchanged. However, we are the first to report that prior exposure of murine BMDMs to 4T1-conditioned medium resulted in heightened sensitivity to TLR agonists such as LPS, as demonstrated by increased production of pro-inflammatory TNFα, IL-6 and MCP-1, as well as increased production of reactive nitrogen intermediates and enhanced phagocytic activity. This effect was not unique to macrophages conditioned with 4T1 carcinomas. Macrophages exposed to LLC- or ID8-conditioned media also exhibited increased TNFα and CCL2 production in response to LPS. In contrast, macrophages stimulated with media conditioned by normal mammary epithelial cells HC11 had no altered sensitivity to LPS treatment. These findings were surprising since it has been shown that TAMs typically exhibit M2-like polarization[4], which elicits decreased inflammatory cytokine production and heightened IL-10 production in response to TLR agonists[20]. Furthermore, a robust TLR-induced inflammatory response seemed unlikely considering the cytokine milieu of the tumor microenvironment, which contains significant amounts of immunosuppressive IL-10 and TGFβ, as well as other anti-inflammatory mediators[2]. However, it must be noted that our model of 4T1-conditioned BMDMs only examines a specific interaction between 4T1-secreted factors and mature macrophages and does not take into account the possible effects of other cell types found in the tumor microenvironment. For example, immunosuppressive B-lymphocytes and regulatory T-cells are themselves sources of IL-10 and TGFβ, as well as M2-polarizing factors such as IL-4[2]. In addition, oxygen tension within the tumor microenvironment has complex effects on macrophage inflammatory functions through the actions of the hypoxia-inducible factors[21]. While the significance of our findings with respect to macrophage behavior in the complex setting of the tumor microenvironment itself remains unclear, there are clear implications regarding the systemic inflammatory state of the host. Since certain tumor-secreted factors are responsible for a transient increase in the inflammatory behavior of BMDMs, it is possible that macrophages distal to the tumor site could be similarly programmed to respond strongly to pathogen-associated molecular motifs. Indeed, we observed that peritoneal macrophages distal to the primary tumors of 4T1-bearing mice produced more inflammatory cytokines in response to LPS stimulation than peritoneal macrophages from non-tumor controls. This phenomenon presents significant consequences in the progression of cancer in the host. Mediators of sterile inflammation, such as HMGB1 or host nucleic acids released from necrotic cells, also initiate immune responses through activation of the TLR or inflammasome signaling pathways[22]. For example, breast cancer patients often present with increased levels of circulating TNFα and IL-6, suggesting a heightened inflammatory state that correlates with hastened metastatic progression and death[23,24]. This is further established in mouse models of breast cancer in which ablation of TNFα or IL-6 results in reduced metastasis[25,26]. While the secretion of these cytokines from the primary tumor undoubtedly plays a role in metastatic progression, our observations strongly suggest that priming of distal macrophages can result in secondary production of TNFα, IL-6, and CCL2. It would be of great interest to investigate whether these 4T1-programmed macrophages play a role in shaping pre-metastatic sites.

Our observations also have important implications for the use of inflammatory agonists in the treatment of breast cancers. TLR agonists as therapeutic agents against cancers have been considered because of their potential to reverse immune suppression within the tumor microenvironment and thereby enhance the Th1-cell-mediated induction of cytotoxic T lymphocytes that are necessary for effective anti-cancer immunity[12–14]. However, the use of pathogen-derived agents in the treatment of cancers has met with limited successes. For example, use of Bacillus Calmette-Guérin (BCG) as an immunotherapeutic agent is only effective against bladder carcinomas [10]. Similarly, the use of LPS-derivative MPL for TLR4 stimulation and TLR7 agonist imiquimod has been restricted to vaccine formulations against HPV-associated cervical cancers and to topical treatments for basal-cell skin carcinomas, respectively[10]. Despite their limited uses at present, there is a strong drive to develop novel synthetic TLR agonists for a wide range of clinical applications, notably within therapeutic vaccine formulations as an immunostimulatory adjuvant, or as a supplement to chemotherapy and radiotherapy [10,27]. However, the potential beneficial applications of these agonists must be viewed with caution in light of numerous studies demonstrating the tumor-promoting effects of TLR ligands[28]. Considering our findings with the 4T1 mammary carcinoma line, it is necessary to consider the possibility that use of TLR agonists in the treatment of certain breast cancers may elicit a significantly enhanced systemic inflammatory reaction, resulting in immunotoxicity and/or tumor progression.

We also sought to elucidate the mechanism by which 4T1-secreted factors alter macrophage inflammatory behavior. The surface expression levels of macrophage markers on 4T1-conditioned BMDMs were examined since macrophage inflammation often correlates with the expression of certain surface receptors[29]. Surprisingly, the surface expression of class II MHC molecules and CD80, which is often elevated in inflammatory macrophages and increased with M1-like polarization[29], did not significantly change with 4T1-conditioning. TLR4 expression was also unchanged, indicating that the increased response to LPS by 4T1-conditioned BMDMs was not simply due to an overexpression of the LPS-receptor. Curiously, surface expression of the classical murine macrophage marker F4/80 was greatly elevated as a result of 4T1-conditioning. While the role of F4/80 with regards to macrophage inflammatory phenotypes is unclear, F4/80 expression has been linked to the suppression of antigen-specific immunity and immune tolerance[30]. Lin *et al* demonstrated that macrophage F4/80 expression is important in the development of regulatory CD8^+^ T-cells, potentially through its role in promoting physical interactions between macrophages and T-lymphocytes[30]. Perhaps the enhancement of macrophage F4/80 expression by 4T1-secreted factors is one of the mechanisms through which this aggressive mouse mammary carcinoma can suppress tumor-specific immune responses[31].

We also investigated the potential role of pro-inflammatory signaling pathways that may be triggered by 4T1-secreted factors through the use of pathway-specific inhibitors. 4T1 cells are a rich source of inflammatory mediators, including macrophage-activating factors, growth factors, and cytokines[15,17]. Carcinomas have also been reported to activate TLR-mediated signaling in macrophages through the release of extracellular matrix proteins [32]. We found that that 4T1-secreted M-CSF played a partial role in priming BMDM responses to bacterial agonists; however, we could find no role for JAK-STAT, phosphatidylinositol 3-kinase, or mitogen-activated protein kinase activating cytokines in the 4T1-mediated regulation of macrophage function, whereas NFκB inhibition showed a strong abrogation of the 4T1 priming effect. Interestingly, inhibition of actin polymerization also prevented 4T1-mediated enhancement of BMDM responses, indicating the importance of actin-mediated uptake of 4T1 factors. This observation is likely indicative of macrophage endocytosis of microvesicles or exosomes, which are released in abundance by many carcinomas[33]. Indeed, we demonstrated that extracellular vesicles are present in 4T1-conditioned medium. Furthermore, disruption of these vesicles resulted in reduced responses of conditioned BMDMs to inflammatory stimuli, indicating that these vesicles played at least a partial role in priming macrophage-mediated inflammatory responses. However, we do not yet know which vesicle-packaged factors were responsible for augmenting BMDM responses to LPS and other inflammatory stimuli. Breast carcinomas are known to exert distal inflammatory effects through numerous regulatory factors, including cytokines and other proteins, as well as RNAs and miRNAs, which are compartmentalized into vesicles[33]. Exosomal RNAs can extensively regulate the gene expression profile on target cells to induce pro-angiogenic and invasion-promoting behaviors in target cells, as well as altering host immune responses[33]. RNA- or miRNA-mediated regulation, rather than cytokines, may be responsible for 4T1 priming of a macrophage inflammatory response, thus explaining the ineffectiveness of many pathway inhibitors in abrogating the phenomenon. The release of exosomes by cancer cells is thought to play a major role in shaping the pre-metastatic niche at distant locales[33]. For example, a recent study shows that systemic administration of breast cancer exosomes to mice results in inflammatory cytokine production by lung macrophages through the transmission of TLR2-activating molecules[18]. Although we see no direct activation of macrophage inflammation by 4T1-secreted factors in our study, similar mechanisms may be involved in priming macrophages for an enhanced inflammatory response. It is interesting to note that the in vitro enhancement of LPS-induced IL-6 and TNFα production seemed to be a transient effect which faded over 72 h, although peritoneal macrophages from 4T1 tumor-bearing mice also exhibited increased production of pro-inflammatory cytokines. Whether this in vitro transiency stems from a tolerance of macrophages to 4T1-secreted factors or whether it reflects the consumption and degradation of these factors is currently unknown. In the former scenario it is feasible that, while mature macrophages may be tolerant to 4T1-secreted molecules, newly generated macrophages or circulating monocytes may be sensitive to the influence of 4T1 tumors, resulting in a constant population of macrophages responding more strongly to inflammatory insult. Newly differentiated tissue macrophages would also be generated in greater numbers during situations of acute inflammation, such as during infection or injury, thus exacerbating the inflammatory state of a cancer bearing host. It must also be noted that 4T1 conditioning of BMDMs exhibits a transient increase in LPS-induced IL-6 and TNFα production, whereas the increase in LPS-induced CCL2 production seems to be persistent. Perhaps this phenomenon is related to the ability of 4T1-conditioned culture media to induce macrophage CCL2 production without LPS intervention. It may be possible that the 4T1 factors which directly induce CCL2 production synergizes with the heightened macrophage sensitivity to inflammatory agonists, thus leading to a prolonged elevation of CCL2 levels. This observation further reinforces the idea that the enhancement of macrophage sensitivity by 4T1 tumors is highly complex and likely mediated by a wide range of secreted factors, each with their own specific behaviors.

In summary, we demonstrate that exposure to 4T1-secreted factors, partially via actin-mediated uptake of exosomes, lead to enhanced macrophage responses to LPS and other bacteria-derived TLR agonists, resulting in increased pro-inflammatory cytokine production, phagocytosis, and nitric oxide release. This phenomenon may have significant implications regarding the potential role of distal macrophages in the metastatic progression of breast cancer. Furthermore, our observations suggest caution for the use of TLR agonists as a component of immune-based treatments for breast cancer.

## Materials and Methods

### Animals

Adult (6- to 8-week old) female C57BL/6 and BALB/c mice purchased from Charles River Canada (Lasalle, QC, Canada) were housed in the Carleton Animal Care Facility and were maintained on a diet of rodent chow and water *ad libitum*. Animal use for this study was approved by the Dalhousie University Committee on Laboratory Animals and was in accordance with Canadian Council of Animal Care guidelines.

### Reagents

LPS from *E*. *coli* 0111:B4 was purchased from Sigma-Aldrich Canada (Oakville, ON, Canada). Type B CpG ODN, flagellin from *S*. *typhimurium*, and peptidoglycan from *E*. *coli* 0111:B4 were obtained from Invivogen (San Diego, CA, USA). Actin polymerization inhibitor cytochalasin D, CSF-1R inhibitor GW2580, p38 inhibitor SB203580, phosphatidylinositol 3-kinase inhibitor wortmannin, microtubule polymerization inhibitor nocodazole, NFκB inhibitor Bay 11–7082, MEK inhibitor PD184352, JNK inhibitor II, and JAK2 inhibitor AG490 were all obtained from Sigma-Aldrich Canada. Ethylenedinitrilotetraacetic acid, disodium salt dehydrate (EDTA) was obtained from MERCK (Darmstadt, Germany). Bovine serum albumin (BSA) and Dulbecco’s phosphate-buffered saline (PBS) were obtained from Sigma-Aldrich Canada. Trypsin-EDTA was obtained from Life Technologies (Burlington, ON). IRF-1 and control siRNA pools, and siRNA transfection reagent were all obtained from (Santa Cruz Biotechnology, Texas, USA).

### Cell culture and conditions

Mouse L929 fibroblasts, 4T1 murine mammary carcinomas, and murine LLC cells were obtained from ATCC (Manassas, VA, USA). E0771 murine mammary carcinoma cells[34], ID8 ovarian carcinoma cells[35], and HC11 mouse mammary epithelial cells [36] were kind gifts from Drs. David Waisman, Craig McCormick, and Hyo Sung Ro (all from Dalhousie University, NS, Canada) respectively. All cell lines were grown in T75 tissue culture flasks (Sarstedt, QC, Canada) and maintained at 37°C and 5% CO_2_ in DMEM containing 10% (v/v) fetal calf serum (FCS), 100 U/ml penicillin, 100 μg/ml streptomycin, 2 mM L-glutamine, and 5 mM HEPES (ph 7.4), hereafter referred to as “DMEM” unless otherwise indicated. At 90% confluence, cells were passaged by detachment with trypsin-EDTA, followed by centrifugation at 500 x *g* for 5 and then resuspension in fresh DMEM. Freshly isolated BMDMs were cultured at 37°C at 5% CO_2_ in RPMI-1640 medium containing 10% FCS (v/v), 100 U/ml penicillin, 100 μg/ml streptomycin, 2 mM L-glutamine, and 5 mM HEPES (ph 7.4), hereafter referred to as “RPMI-1640” unless otherwise indicated. Prior to experimental use, BMDMs were detached with EDTA (10 mM in PBS), centrifuged at 500 x *g* for 5 min, and resuspended in fresh RPMI-1640 before cell seeding.

### Isolation of BMDMs

BMDMs were generated as previously described [37]. Briefly, bone marrow cells were obtained by flushing the femurs and tibias of mice with PBS. Red blood cells were removed through osmotic lysis and the bone marrow cell suspension was washed twice with PBS, then cultured at 1 x 10^6^ cells per well in 6-well tissue culture plates (Thermo Scientific, Rochester, NY, USA) with BMDM medium consisting of RPMI-1640 medium containing 10% FCS (v/v), 100 U/ml penicillin, 100 μg/ml streptomycin, 2 mM L-glutamine, 5 mM HEPES (pH 7.4), and 15% L929-conditioned DMEM (v/v) as a source of M-CSF. After 3 days of culture, the cells were fed with fresh BMDM medium. At day 6, culture medium and non-adherent cells were removed and the remaining adherent cells were fed with fresh BMDM medium. At day 7, BMDM purity was greater than 90%, as determined by flow cytometric measurement of the surface expression of macrophage marker F4/80 (FACSCalibur, BD Bioscience, Mississauga, ON, Canada).

### Generation of tumor-conditioned medium and BMDM conditioning

Murine carcinoma cells and HC11 mammary epithelial cells were seeded at 2.5 x 10^5^ and 5 x 10^5^ cells per T75 tissue culture flask (Sarstedt), respectively, in DMEM containing 10% FCS (v/v), 100 U/ml penicillin, 100 μg/ml streptomycin, 2 mM L-glutamine, and 5 mM HEPES (pH 7.4) (Invitrogen, Burlington, ON, Canada). After 4 days of culture and at 90% confluence, medium was collected and passed through a Filtropur syringe filter membrane (0.2 μm pore, Sarstedt). Filtered cell-free medium was stored at -20°C until use.

For conditioning, BMDMs were seeded onto 24-well plates (Thermo Scientific) at 2.5 x 10^5^ cells per well in RPMI-1640 and incubated for 4 h at 37°C to allow for adherence. Culture medium was then removed and the cells were washed with PBS before the addition of fresh RPMI-1640 and 10% (v/v) tumor-conditioned DMEM. BMDMs were then cultured in this medium for 24 h, unless otherwise indicated. BMDM cultures containing 10% DMEM (v/v) or 10% HC11-conditioned medium (v/v) were used as non-conditioned and non-tumor controls, respectively.

### Chemotaxis assay

BMDMs were seeded at 2.5 x 10^5^ cells per well on 24-well plates in RPMI-1640 and allowed to adhere for 4h. Cells were then treated with tumor-conditioned culture medium for 24 h and the supernatants collected. Supernatants were then placed in the bottom wells of a 48-well micro chemotaxis chamber with the upper wells containing freshly isolated BMDMs in RPMI-1640 with 0.1% BSA (w/v) at 2 x 10^4^ cells per well (Neuroprobe, Gaithersburg, MD, USA). In CCL2-neutralization studies, BMDM supernatants were pre-treated for 1h with 10 μg/ml of a goat IgG anti-mouse CCL2 antibody (R&D Systems, Minneapolis, MN, USA) or a goat IgG isotype control antibody (Sigma-Aldrich) prior to their use in the chemotaxis assays. To control for pre-existing chemokines produced by tumor cells, tumor-conditioned media alone was added to the lower wells. Lower wells containing only RPMI-1640 with 0.1% BSA (w/v) were used as vehicle controls. The upper and lower wells were separated by a polycarbonate membrane with 5 μm pores. The chamber was incubated at 37°C for 4 h to allow for migration of BMDMs. Each treatment condition was performed in quadruplicate. After incubation, non-migrated cells were removed by washing one side of the membrane with PBS and scraping off cells with a rubber blade. The membrane, containing only migrated BMDMs, was stained using the Diff-Quik staining kit (Siemens Healthcare Diagnostics Inc., Newark, DE, USA) following manufacturer’s protocols and mounted onto a glass microscope slide. Migration was assessed by averaging the number of cells per field of five randomized high-powered fields (200x magnification). For each treatment, the values of the four replicates were again averaged. Relative migration was calculated by taking the migrated cells per high power field of each treatment and comparing it against the migrated cells per high power field of the vehicle control, yielding fold change-over-control values.

### Determination of chemokine gene expression by real-time quantitative PCR

BMDMs were seeded at 1 x 10^6^ cells per well into 6-well plates in RPMI-1640,allowed to adhere for 4h, and then conditioned, as described above, with 4T1- or HC11-conditioned DMEM for 24 h. The cells were lysed and total RNA was obtained using the Qiagen RNeasy Mini kit (Toronto, ON, Canada), following the manufacturer’s protocols. RNA concentration was assessed using a NanoDrop spectrophotometer and 500 ng of total RNA was reverse transcribed using SuperScript II Reverse Transcriptase enzyme (Invitrogen, Burlington, ON, Canada) following manufacturer’s protocols. Real-time PCR was performed in a 10 μl reaction, with 1 μl of 1/10 diluted cDNA, using the FastStart SYBR Green Master kit (Roche, Mississauga, ON, Canada), following manufacturer’s instructions. Melting curves were performed to verify the production of specific amplicons. The primer sequences against the coding sequence of each chemokine (Integrated DNA Technologies, Coralville, IA, USA) are presented in Table 1. Changes in gene expression were calculated by normalizing the change in expression of each gene of interest to that of housekeeping gene GAPDH using the delta-Ct method. Gene expression of each chemokine by conditioned BMDMs was then normalized to that of non-conditioned BMDMs, yielding fold change-over-control values.

**Table 1:** Primer sequences for real-time PCR.

Gene	Forward	Reverse
CCL2	AACTCTCACTGAAGCCAGCTCT	CGTTAACTGCATCTGGCTGA
CCL4	TGTGCAAACCTAACCCCGAG	TGGAGCAAAGACTGCTGGTC
CCL5	CCTCACCATATGGCTCGGAC	ACGACTGCAAGATTGGAGCA
GAPDH	TGCCCCCATGTTTGTGATG	TGTGGTCATGAGCCCTTCC
KC	CAATGAGCTGCGCTGTCAGTG	CTTGGGGACACCTTTTAGCATC

### Determination of cytokine production

To investigate the baseline cytokine production by conditioned macrophages, BMDMs were seeded into 24-well plates 2.5 x 10^5^ cells per well in RPMI-1640,allowed to adhere for 4 h, and then treated with 10% 4T1- or HC11-conditioned DMEM (v/v) for 24 h. Culture supernatants were collected and levels of TNFα, IL-6, and CCL2 were measured by sandwich enzyme-linked immunosorbent assay (ELISA) using commercial kits (eBioscience, San Diego, CA, USA), following manufacturer’s protocols. To account for pre-existing cytokines in the tumor-conditioned media, ELISAs were also performed on 10% 4T1- and HC11-conditioned DMEM (v/v) in the absence of macrophages. Recombinant murine cytokines (Peprotech, QC, Canada) were used to generate protein standard curves.

To investigate cytokine production by conditioned macrophages responding to bacterial agonists, BMDMs at 2.5 x 10^5^ per well in 24-well plates were treated with 10% tumor-conditioned DMEM for 24 h,then washed with PBS and cultured in RPMI-1640 with bacterial agonists for the indicated period of time. Culture supernatants were collected and levels of TNFα, IL-6, IL-10 and CCL2 were determined by ELISA, following manufacturer’s instructions (eBioscience). In BMDM tumor-conditioning time-course studies, tumor-conditioned DMEM was diluted to 10% (v/v) in BMDM medium, instead of RPMI-1640, to prevent macrophage death. In studies using chemical inhibitors, BMDMs were first pre-treated for 1 h with inhibitors at the indicated concentrations, prior to tumor-conditioning. Inhibitor concentrations were also maintained throughout the 24 h tumor-conditioning period. The concentration of DMSO in cultures never exceeded 0.1% (v/v) and DMSO at this concentration was used as a vehicle control for all inhibitor studies. As above, after conditioning, culture medium was removed and BMDMs were washed with PBS prior to stimulation with bacterial agonists. In studies using vesicle-cleared medium, a Sonic Dismembrator 60 (Fisher Scientific, ON, Canada) was used to sonicate tumor-conditioned medium by pulsing with the sonicator probe for 5 x 10s on/off cycles. Samples were kept on ice during sonication to prevent significant heating. Vesicle-cleared medium were then diluted in RPMI-1640 to 10% (v/v) prior to the treatment of BMDMs for 24 h. As before, culture medium was removed and BMDMs were washed with PBS prior to stimulation with LPS for 4h. Supernatants were then collected and cytokine levels were measured by ELISA. Finally, in studies investigating the siRNA-mediated knockdown of IRF-1, BMDMs were transfected with pools of IRF-1 or control siRNA at the indicated concentrations for 24 h according to manufacturer’s protocols (Santa Cruz Biotechnology). BMDMs were then washed with PBS prior to a 24 h stimulation with tumor-conditioned media. Cells were again washed with PBS and stimulated with LPS for 4 h.

### Nitric Oxide Assay

BMDMs were seeded at 2.5 x 10^5^ cells per well into 24-well plates in RPMI-1640, allowed to adhere for 4 h and then treated with tumor-conditioned culture medium for 24 h. After tumor conditioning, BMDMs were stimulated with the indicated concentrations of bacterial agonists for 24 h. Culture supernatants were collected and 100 μl of supernatant was combined with 100 μl of modified Griess reagent (Sigma-Aldrich) in a 96-well flat-bottom plate (Thermo Scientific) and allowed to incubate for 5 min. Nitric oxide levels for each condition was measured by reading the absorbance at 570 nm with a ASYS Expert 96-well plate reader (Montreal Biotech Inc., Dorval, QC, Canada) and comparing it with absorbance values of known concentrations of sodium nitrite (Sigma-Aldrich) in PBS.

### Hydrogen Peroxide Assay

BMDMs were treated with tumor-conditioned culture medium for 24h, then washed and resuspended in RPMI-1640 without phenol red. BMDMs were allowed to adhere onto a 96-well flat-bottom plate at 5 x 10^4^ cells per well, then were stimulated with the indicated bacterial agonists for 24 h at 37°C in the presence of Amplex Red reagent and horseradish peroxidase (Life Technologies), in accordance with the manufacturer’s protocols. Levels of hydrogen peroxide for each condition were measured by reading the absorbance at 570 nm of and comparing it with absorbance values of known concentrations of hydrogen peroxide.

### Macrophage phagocytosis

BMDMs were seeded at 2.5 x 10^5^ cells per well into 24-well plates in RPMI-1640, allowed to adhere for 4h, and then treated with tumor-conditioned culture medium for 24 h. Following a wash with PBS, conditioned BMDMs were incubated at 37°C with 2 x 10^5^ pHrodo Green *E*. *coli* BioParticles resuspended in RPMI-1640 (Life Technologies) for 2 h. Non-phagocytosed bioparticles were removed by two rounds of washing with PBS. Cells were then detached by EDTA (10mM in PBS), centrifuged for 5 min at 500 x *g*, and resuspended in PBS containing 1% BSA (w/v). The fluorescent emission of internalized bioparticles was measured by flow cytometry. As a negative control, BMDMs were pre-treated for 1 h with actin polymerization inhibitor cytochalasin D (10 μM) prior to incubation with *E*. *coli* bioparticles. For each treatment condition, the fluorescence level of 10,000 macrophages was obtained and the geometric mean fluorescence intensity was calculated by CellQuest (BD Bioscience). Percent phagocytosis was calculated by comparing the mean fluorescence intensity of each experimental value to that of non-conditioned macrophages.

### Fluorescent staining of macrophage surface markers

BMDMs were seeded at 2.5 x 10^5^ cells per well into 24-well plates in RPMI-1640, allowed to adhere for 4h, and then treated with tumor-conditioned culture medium for 24 h. Following a wash with PBS, conditioned BMDMs were detached by EDTA (10mM in PBS), centrifuged for 5 min at 500 x *g*, and washed with PBS containing 1% BSA (w/v). BMDMs were then stained for 30 min with a PE-conjugated rat IgG2a anti-mouse F4/80 antibody (Ab) (Clone BM8, eBioscience), a PE-conjugated rat IgG2b anti-mouse CD11b Ab (Clone M1/70, Biolegend, San Diego, CA, USA), a PE-conjugated rat IgG2a anti-mouse TLR4 Ab (Clone SA15-21, Biolegend), a FITC-conjugated hamster IgG2 anti-mouse CD80 Ab (Clone 16-10A1, eBioscience), a FITC-conjugated rat IgG2a anti-mouse CD40 Ab (Clone 3/23, Biolegend), a FITC-conjugated rat IgG2a anti-mouse CD206 Ab (Clone C068C2, Biolegend), or a FITC conjugated rat IgG2b anti-mouse MHC class II (I-A/I-E) (Clone M5/114.15.2, eBioscience). A PE-conjugated rat IgG2a Ab (Clone RTK2758, Biolegend), a PE-conjugated rat IgG2b Ab (Clone RTK4530, Biolegend), a FITC-conjugated rat IgG2b Ab (Clone eB149/10H5, eBioscience), or a FITC-conjugated hamster IgG2 Ab (Clone eBio299Arm, eBioscience) were used as isotype antibody controls. Following staining, BMDMs were washed twice with PBS containing 1% BSA (w/v). To determine the levels of macrophage surface markers for each treatment, the fluorescence level of 10000 BMDMs was obtained by flow cytometry. The geometric mean fluorescence intensity was measured by FACSCalibur and calculated by CellQuest.

### Electron microscopy

A drop of 4T1- or HC-11 conditioned medium was placed onto Formvar/carbon-coated grids and allowed to settle for 5 min. Each sample was then stained with a 2% uranyl acetate solution as a negative stain for extracellular vesicles. Following staining, the grids were dried and visualized under a JEM-1230 transmission electron microscope (JEOL USA, Peabody, MA, USA). Samples were visualized under vacuum with an electron beam with an accelerating voltage of 80 000 V. The resulting images are displayed at 80 000x magnification.

### Isolation of peritoneal macrophages from tumor-bearing mice

Adult female BALB/c mice were each injected with 1 x 10^5^ 4T1 murine mammary carcinoma cells (n = 6) or saline (n = 4) into the left mammary fat pad. Primary tumors of 4T1-injected mice were palpable 8 days later and all mice were euthanized 16 days post-injection. Peritoneal macrophages were isolated as described previously [38]. Briefly, peritoneal cells were obtained by flushing the peritoneum with 10 ml of cold PBS and aspirating the liquid. Exudate cells were centrifuged for 5 min at 4°C at 500 x *g*, washed with cold PBS, and centrifuged again. Peritoneal cells were seeded in RPMI-1640 at 5 x 10^5^ cells per well in a 24-well tissue culture plate and allowed to adhere for 2 h at 37°C. Non-adherent cells were washed off with PBS and the remaining macrophages were counted and incubated in RPMI-1640 prior to stimulation with LPS.
